# The Value of a Checklist for Child Abuse in Out-of-Hours Primary Care: To Screen or Not to Screen

**DOI:** 10.1371/journal.pone.0165641

**Published:** 2017-01-03

**Authors:** Maartje CM Schouten, Henk F. van Stel, Theo JM Verheij, Michiel L. Houben, Ingrid MB Russel, Edward ES Nieuwenhuis, Elise M. van de Putte

**Affiliations:** 1 Division of Pediatrics, Wilhelmina Children’s Hospital, University Medical Center Utrecht, Utrecht, the Netherlands; 2 Julius Center for Health Sciences and Primary Care, University Medical Center Utrecht, Utrecht, the Netherlands; Foundation for Medical Research, INDIA

## Abstract

**Objectives:**

To assess the diagnostic value of the screening instrument SPUTOVAMO-R2 (checklist, 5 questions) for child abuse at Out-of-hours Primary Care locations (OPC), by comparing the test outcome with information from Child Protection Services (CPS). Secondary, to determine whether reducing the length of the checklist compromises diagnostic value.

**Methods:**

All children (<18 years) attending one of the participating OPCs in the region of Utrecht, the Netherlands, in a year time, were included. The checklist is an obligatory field in the electronic patient file. CPS provided data on all checklist positives and a sample of 5500 checklist negatives (dataset). The checklist outcome was compared with a report to CPS in 10 months follow up after the OPC visit.

**Results:**

The checklist was filled in for 50671 children; 108 (0.2%) checklists were positive. Within the dataset, 61 children were reported to CPS, with emotional neglect as the most frequent type of abuse (32.8%). The positive predictive value (PPV) of the checklist for child abuse was 8.3 (95% CI 3.9–15.2). The negative predictive value (NPV) was 99.1 (98.8–99.3), with 52 false negatives. When the length of the checklist was reduced to two questions closely related to the medical process (SPUTOVAMO-R3), the PPV was 9.1 (3.7–17.8) and the NPV 99.1 (98.7–99.3). These two questions are on the injury in relation to the history, and the interaction between child and parents.

**Conclusions:**

The checklist SPUTOVAMO-R2 has a low detection rate of child abuse within the OPC setting, and a high false positive rate. Therefore, we recommend to use the shortened checklist only as a tool to increase the awareness of child abuse and not as a diagnostic instrument.

## Introduction

Child abuse is a major global health problem [1;2]. In the Netherlands, the prevalence of child abuse is calculated at 34/1000, with a total of 118.836 children in 2010 [3]. Children who are abused are at higher risk for developing social, behavioral and medical problems [4–6]. Because of these possible long-term effects, early detection of child abuse is essential.

Screening instruments can assist physicians in recognizing child abuse at an early stage, thereby probably preventing any further harm [7;8]. Important places for recognizing child abuse are emergency rooms and Out-of-hours Primary Care locations (OPC) [9;10]. A screening protocol with a screening instrument for child abuse at OPCs is mandatory in the Netherlands since 2011 [11]. One of the advised tools for screening is the SPUTOVAMO checklist. SPUTOVAMO, or the revised version SPUTOVAMO-R, is a screening instrument using various questions to determine whether there is a suspicion of child abuse [9;12]. Sittig et al. assessed the efficacy of detecting child abuse using the SPUTOVAMO-R at emergency rooms, and found low positive predictive values [13]. Louwers et al. showed a moderate sensitivity and specificity of each question in their comparative screening instrument for child abuse, in a selected population (the screening instrument was not mandatory and consequently only filled in for 48% of the children) at emergency departments [8]. Evidently, the use of a screening instrument increases the awareness of child abuse [7]. However, comprehensive evidence on the accuracy of screening instruments for child abuse, and data for OPCs are lacking. Furthermore, most prior research was limited by the lack of approval for verification of the checklist negative results for confirmation of the diagnosis (i.e. child abuse present or absent) [7]. In addition, until now, no diagnostic value of screening instruments has been studied for all types of abuse in each child (unselected population) attending an emergency room or OPC [8;13–16]. For this study, we revised the SPUTOVAMO-R for emergency rooms into SPUTOVAMO-R2 for OPCs.

The primary aim of this study was to assess the diagnostic value of the screening instrument SPUTOVAMO-R2 for child abuse at OPCs, by comparing the test outcomes with information from CPS. The secondary aim was to determine if reducing the length of the SPUTOVAMO-R2 is possible without compromising the diagnostic value.

## Methods

### Study population

All children aged 0–18 years attending one of the five participating OPCs in the region Utrecht, the Netherlands, between July 2012 and July 2013 were included. All the OPCs in this region are unified in one organization (Primair Huisartsenposten).

### Checklist

**Fig 1:**
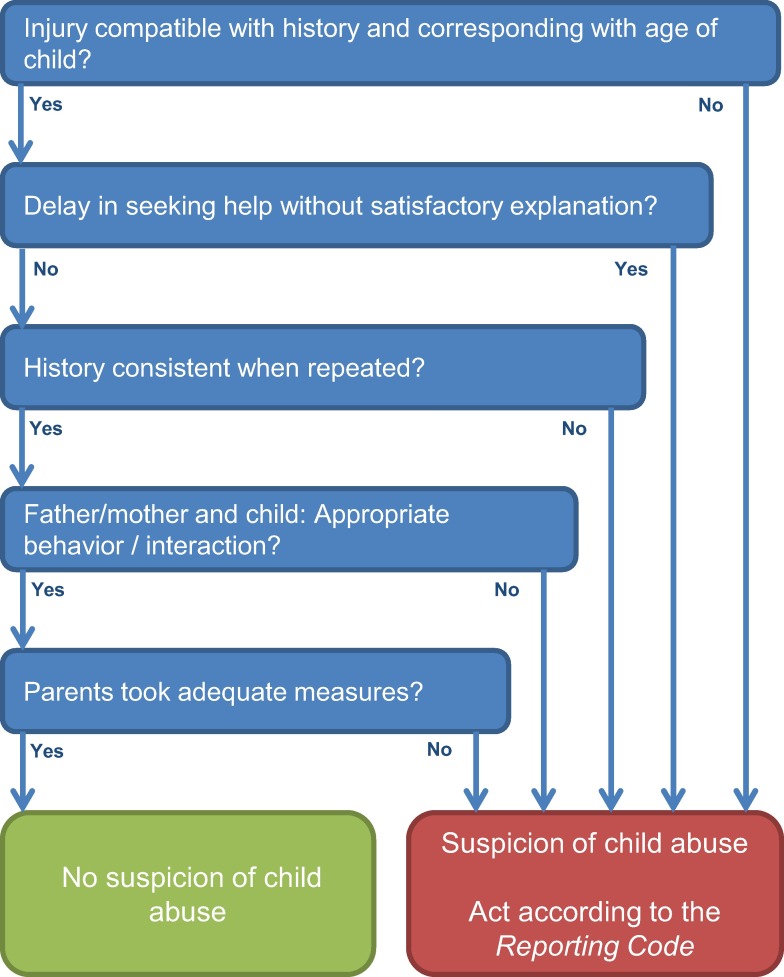
SPUTOVAMO-R2 screening instrument for child abuse. *Reporting code*: In the Netherlands, in case of a suspicion of child abuse, medical doctors are obligated to act according to the five steps of the reporting code for child abuse [17]. This reporting code ensures a thorough diagnostic process and careful communication with patient and family. With consistent use of the steps, a sound decision on whether to report to child protection services can be reached.

The screening instrument SPUTOVAMO-R2 is the second revised version of the original [12], to which we will further refer as the checklist. The checklist consists of five questions (Fig 1). If one question is answered deviant, the checklist classifies positive for suspected child abuse. At OPCs, the checklist is filled in for all patients under the age of 18; it is an obligatory field in the electronic patient file.

### Procedure

All children were screened for child abuse using the checklist. The checklist was filled in at the end of the consultation by the general practitioner at the OPC. In case of a positive checklist result, the child received clinical care according to regional and national guidelines, independently of this study [17–19]. According to the regional guideline at the OPCs, each child with a positive checklist result was evaluated in a multidisciplinary group, to decide about necessary care for this child and family.

In the absence of a gold standard for the diagnosis of child abuse, a report to CPS was chosen as the reference standard. In the Netherlands, the physician has ‘the right’ but not the obligation to report to CPS.

In July 2014, CPS provided on request data on all checklist positives and a sample of 5500 checklist negatives. A minimum sample of 4067 checklist negatives was calculated to be necessary, based on a sensitivity of 90% and a confidence level of 95% [8;13]. We estimated that around 4200 children are monthly seen at the five OPCs, resulting in an inclusion period of one month for all checklist negatives. CPS provided data on whether the child was reported to CPS in the 10 months following the OPC visit; and in case of CPS report, the profession of the reporter and the type of abuse was noted.

### Reference standard

The checklist outcome was tested against a report to CPS in 10 months follow up after attending the OPC. The exact date of attending the OPC was available for all checklist positives. The sample of checklist negatives attended the OPC in the month February 2013, resulting in a 10 month follow up period ending in December 2013. CPS was not informed on the outcomes of the checklist.

### Ethical approval

All children and parents attending the OPCs were informed about the study and the standard clinical care by a flyer, which was handed over to them. Privacy protection was cared for with a trusted third party (CPS), to guarantee the privacy and anonymity of each child. The data collected at the OPC were transferred to CPS. CPS performed the match between OPC data and CPS data with identifiable patient information (name and date of birth). After their data acquisition, they extracted the patient name and transformed the date of birth into age (in years). The researcher received these anonymized. According to the Dutch Medical Research Involving Human Subjects Act, this kind of diagnostic study is exempt from ethical review (confirmed by the Medical Ethical Review Board, UMC Utrecht, protocol number 12-286/C).

### Patient involvement

No patients were involved in the development and design of the study, nor were they involved in the implementation of the study.

### Statistical analyses

Characteristics of the children were described using median and percentages. The annual prevalence of child abuse at OPCs was calculated with extrapolation of the CPS reports in the checklist negative sample (n = 5500) to the total population checklist negative OPC minor visitors, and with summation of the confirmed checklist positives, divided by the total number of minor OPC attendances. All predictive values, sensitivity and specificity were calculated with 95% binomial exact confidence intervals (CI). Those values were also calculated with extrapolation of the sample to the total population OPC minor visitors. Risk factors were analyzed using logistic regression and results expressed as odds ratios (OR) for child abuse. An adapted checklist was developed based on diagnostic values, odds ratios, and clinical applicability. The adapted checklist was validated within the same participant data.

Detection rates were defined as the proportion of cases—determined by the reference standard—detected by the checklist. The detection rates were calculated as follows: the number of CPS reports in the checklist positives, divided by the number of CPS reports in the checklist negatives (n = 5500) extrapolated to the total population of checklist negative OPC minors with summation of the confirmed checklist positives.

Differences were considered statistically significant if 2-sided *p* was less than 0.05. All analyses were performed using SPSS version 21.

## Results

### Patient flow and baseline characteristics

**Fig 2:**
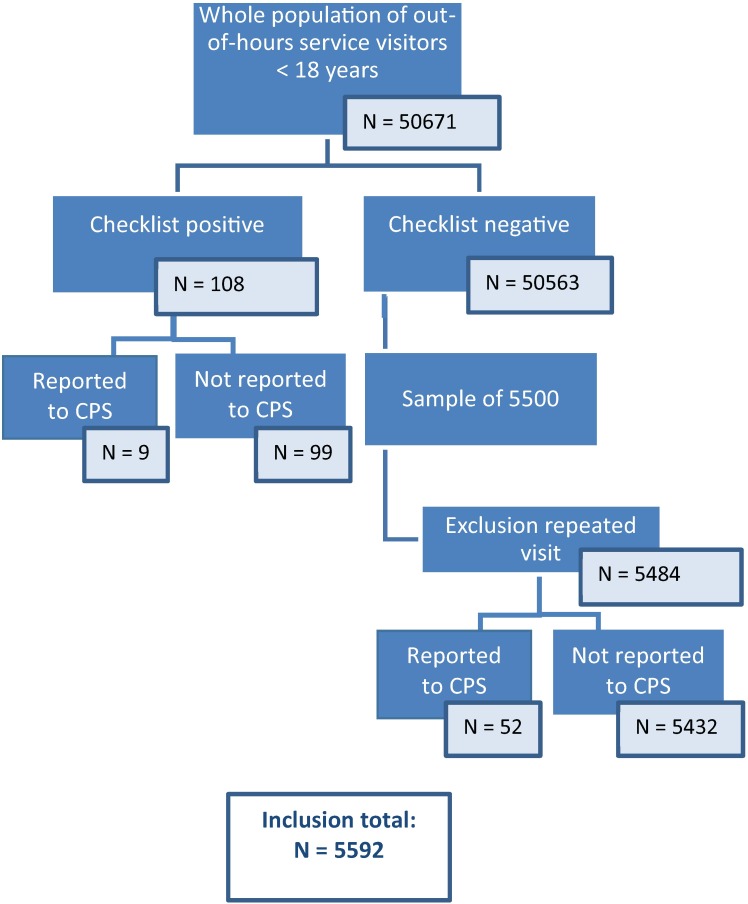
Flow diagram of screening for child abuse in the five Out-of-hours Primary Care locations.

A total of 50671 children were screened with the checklist in a year time. Of these, 108 (0.2%) had a positive checklist. With a sample of 5484 children with a negative checklist, data on 5592 children were analyzed (Fig 2 and S1 and S2 Datasets). No adverse events were reported to the general practitioners or the OPCs by children or their families, from filling in the checklist for all children. The median age was 3.0 (IQR 1–8) years; 54% were male.

For two children, information on which questions were answered deviant on the checklist were missing.

### Prevalence of child abuse

In a year time, 61 children were reported to CPS. The annual prevalence of child abuse–as determined by the reference standard—at the OPCs was calculated at 1.0% (with correction for repeat visitors).

### Diagnostic value of the checklist

Table 1 shows the diagnostic value of the checklist for child abuse. The positive predictive value (PPV) of the checklist was 8.3 (95% CI 3.9 to 15.2), with 99 false positives. The negative predictive value (NPV) was 99.1 (95% CI 98.8 to 99.3), with 52 false negatives. With the exception of smaller confidence intervals, the same diagnostic value was found with extrapolation of the sample to the total population OPC minor visitors, with 478 false negatives. The detection rate of the checklist SPUTOVAMO-R2 for child abuse at OPCs was 9/487 = 1.8%.

**Table 1:** Value of the checklist SPUTOVAMO-R2 for diagnosis of child abuse reported to CPS in 10 months follow up.

**Index test**		Reference test: Report to CPS		
		Yes	No	Total
**Checklist**	Positive	9	99	108
	Negative	52	5432	5484
	Total	61	5531	5592
	PPV	8.3 (3·9–15·2)		
	NPV	99.1 (98·8–99·3)		
	Sens	14.8 (7·0–26·2)		
	Spec	98.2 (97·8–98·5)		

CPS = child protection services; PPV = positive predictive value (+ 95%CI); NPV = negative predictive value (+ 95%CI); Sens = sensitivity (+ 95%CI); Spec = specificity (+ 95%CI).

### Type of abuse

**Fig 3:**
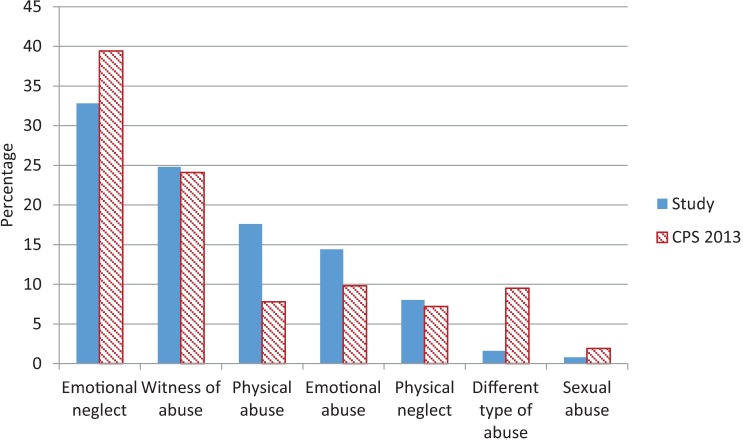
Type of abuse (%) reported to CPS in 10 months follow up in 61 study cases versus type of abuse (%) reported to CPS in the Netherlands in the year 2013 (n = 33571).In 38 study cases there were multiple types of abuse reported.

**Fig 4:**
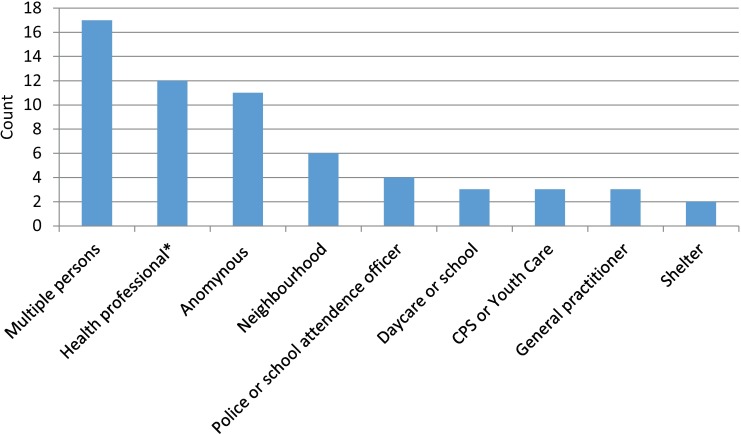
Person reporting the child to CPS in 10 months follow up, in 61 cases.

Emotional neglect (32.8%) and being a witness of abuse (24.8%) were the most frequent types of child abuse reported to CPS. Thirty-eight children were victim of multiple types of abuse (Fig 3). Of the 61 children reported to CPS, three were reported by a general practitioner (Fig 4). Of the nine checklist positive children, five were diagnosed with physical abuse.

### Risk factors associated with child abuse

Checklist positives had significantly higher odds for child abuse reported to CPS than the checklist negatives (OR 9.5, 95% CI 4.6 to 19.8; p <0.0001).

### Adapted checklist

**Fig 5:**
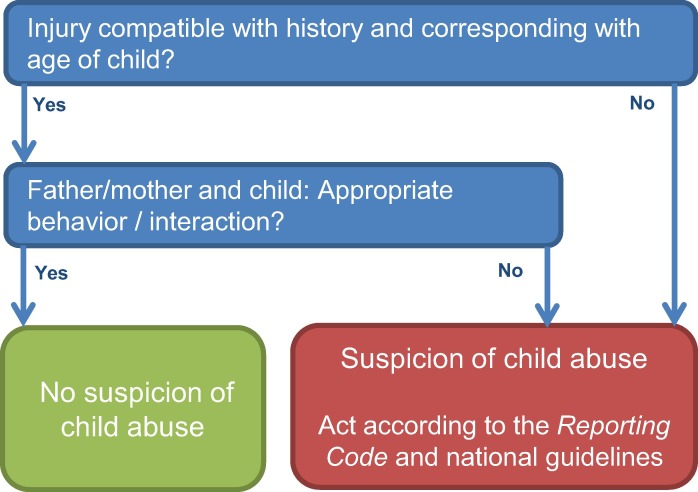
Adapted checklist SPUTOVAMO-R3.

Post hoc, we developed an adapted checklist (SPUTOVAMO-R3) which consisted of two questions: ‘Is the injury compatible with history and corresponding with age of child?’ and ‘Is there appropriate behavior/interaction between father/mother and child?’ (Fig 5). Table 2 shows the diagnostic value of the adapted checklist. The PPV of the adapted checklist for child abuse reported to CPS in 10 months follow up was 9.1 (95% CI 3.7 to 17.8), with 29 less false positives (70 versus 99). The NPV was 99.1 (95% CI 98.7 to 99.3), with one more false negative (53 versus 52). The detection rate of child abuse by the checklist SPUTOVAMO-R3 at OPCs was 7/494 = 1.4%.

**Table 2:** Value of adapted checklist SPUTOVAMO-R3 for the diagnosis of child abuse reported to CPS in 10 months follow up.

**Index test**		Reference test: Report to CPS		
		Yes	No	Total
**SPUTOVAMO-**	Positive	7	70	77
**R3**	Negative	53	5460	5513
	Total	60^a^	5530^a^	5590^a^
	PPV	9.1 (3·7–17·8)		
	NPV	99.1 (98·7–99·3)		
	Sens	11.7 (4·8–22·6)		
	Spec	98.7 (98·4–99·0)		

CPS = child protection services; PPV = positive predictive value (+ 95%CI); NPV = negative predictive value (+ 95%CI); Sens = sensitivity (+ 95%CI); Spec = specificity (+ 95%CI).

^a^ For two children, information on which questions were answered deviant on the checklist missed.

## Discussion

Child abuse is a major health concern, and not one single case should be missed. Therefore, a screening instrument which can definitely rule out child abuse is preferable. Despite the high NPV of the checklist SPUTOVAMO-R2 (99.1%), the majority of cases are still missed. It is possible to reduce the length of the checklist to two questions, and maintain a comparable diagnostic value. The question is however, whether the low detection rate of the checklist—even if it consists of only two questions—justifies implementation.

To our knowledge, this is the first study assessing the diagnostic value of a screening instrument for all types of abuse among all children attending an OPC or emergency room. Before we discuss implementation, some limitations need to be discussed. Firstly, a report to CPS in 10 months follow up after the OPC visit was set as reference standard for child abuse. With ‘the physician’s right’ but not the obligation to report to CPS, cases might have been missed. On the contrary, not all CPS reports (our reference standard) are cases. In general, around 7% of CPS reports are rejected as child abuse after further investigation [20]. Secondly, with a 10 months follow-up period, it is questionable whether child abuse as reported to CPS was necessarily associated with the complaint/injury for which the child visited the OPC. Hence, it is possible that at the moment of the OPC visit, signals and symptoms of child abuse were lacking. Because of privacy reasons, it was impossible to check the medical files of the children who attended the OPC for the presenting symptoms. Lastly, the use of a multidisciplinary group to assess necessary care for all checklist positive children might have led to extra care instead of reporting to CPS. As said, in the Netherlands, physicians have the option to implement care when there are safety issues which possibly can be averted [17;19]. Despite these limitations and consequently possible misclassification in both ways, substantial influence on our main findings is unlikely.

The prevalence of 1.0% found in our study, is lower than the 3.4% calculated by Alink et al. [3]. This could be due to the different reference standard for child abuse; Alink et al. calculated the prevalence based on a summation of CPS reports and concerns by professionals (not necessarily CPS reports). The estimated prevalence of CPS reports in the region of Utrecht in 2012/2013 (all ages) was 0.84% (Netherlands Youth Institute, information on request, 2015), which is close to the prevalence we found. Comparing the types of abuse reported to CPS in our study and in the Netherlands in 2013, we found a comparable distribution (Fig 3). Emotional neglect is the most frequent type of abuse found, which is difficult to recognize for general practitioners at OPCs. At OPCs, the child is often only seen once (and nearly always: once per physician), therefore it is almost impossible to recognize a *repetitive pattern* of abuse, as is particularly characteristic for the diagnosis emotional neglect. A higher percentage of emotional abuse was found for our study population than for the general population in the Netherlands (Fig 3). However, with low numbers of the different types of abuse, the relevance of this finding is disputable. Of the children reported to CPS, only three were reported by a general practitioner. This is probably due to the fact that general practitioners generally refer children with a suspicion of child abuse to the pediatrician for further analysis (unpublished data). According to data from CPS in the Netherlands in 2013, more children were reported by hospital employees (8.5%) than by general practitioners (1.6%)[20].

The finding of a high NPV and a low PPV of the checklist is corroborated by other studies assessing the diagnostic value of screening instruments. The screening instrument ESCAPE, consisting of six questions that are comparable to our checklist questions, was found to have an NPV of 99% and a PPV of 10% for all types of child abuse [8]. The ESCAPE checklist was studied among children presenting at an emergency room. However, without mandatory application of this checklist, selection bias may have occurred. The SPUTOVAMO-R was also studied at an emergency room, but among children less than seven years old. The SPUTOVAMO-R has an NPV of 100% and a low PPV of 3% for non-accidental injury [13]. This high NPV could be reached because the outcome was merely inflicted injury–physical child abuse—present at the moment of the emergency room visit as assessed by an expert panel.

In our study, reducing the length of the checklist to two questions (adapted checklist) is possible, without compromising—but neither clearly improving—the diagnostic value. With this adaption, the detection rate of the checklist remains low.

A total of 50671 children were screened for child abuse with the checklist. Eventually, the checklist only detected 1.8% of the children who were reported to CPS. With this very low detection rate, one could argue about the effectiveness of a checklist for child abuse in such setting [21]. Nevertheless, the checklist can be used without any serious harm for the child and family; no adverse events were reported in this study. We assume that with clear instructions for communication, the burden of an incorrect suspicion can be minimized. The use of the checklist does foresee in recognizing some victims of abuse. With cases often being missed [22], the checklist is therefore of additional value to the physician in recognizing an unsafe situation and intervene. Furthermore, in the study of Sittig et al., the SPUTOVAMO-R had a PPV of 33% for need for help (i.e. ‘any concern about the situation of the child that requires consultation of social services’)[13]. The adapted checklist consists of only two questions, which are highly related to the medical process. These two questions could be part of the assessment of any pediatric clinical problem to ensure the awareness of child abuse, and the responsibility of the physician in diagnosing abuse. With the use of a checklist, registration of this assessment in the file of the child is secured. Registration is then also secured in the file of the child’s own general practitioner, when good communication between the OPC and the child’s own general practitioner exists. Additionally, a positive checklist creates a possibility to start a conversation about safety concerns, with more than just a ‘gut feeling’. We question if it is at all possible to develop a checklist with a NPV of 100% for all types of child abuse. In our opinion, the checklist should therefore be essentially used as a tool to increase the awareness of child abuse; to incorporate child abuse in the differential diagnosis of every child [23]. When the checklist is applied, physicians need to be aware of the limitation of the low accuracy of the checklist and the necessity of diagnostic evaluation in case of a positive checklist to minimize the false positives [13]. Guidelines–with clinical decision rules–for specific injuries, such as fractures, bruises and inflicted brain injury seems more promising in correctly identifying child abuse [24–27]. The use of a checklist should never replace training of physicians, resulting in higher levels of knowledge, confidence and performance [28–30]. This training should focus on symptoms highly suggestive for abuse, communication with child and family, and especially on creating an environment in which a child feels comfortable enough to discuss safety issues.

## Conclusion

In conclusion, the SPUTOVAMO-R2 has a comparable diagnostic value as screening instruments already in use. With the low detection rate of child abuse, we recommend to use a checklist only as a tool for increasing the awareness of child abuse and not as a diagnostic instrument. The use of a checklist guarantees registration of a suspicion in the file of the child, which enables physicians to recognize a possible repetitive pattern. When a checklist is applied, the SPUTOVAMO-R3—with only two questions which are closely related to the medical process—is the most efficient option. In case of a positive checklist, diagnostic evaluation is necessary to confirm or reject a diagnosis of child abuse due to the high number of false positive checklists. Further research on the value and accuracy of guidelines for specific injuries in these care settings is necessary. Before implementation of SPUTOVAMO-R3, careful consideration of the clinical and societal impact is advised and a cost-effectiveness ratio should be assessed.

## Supporting Information

S1 Dataset Dataset. (XLS)

S2 Dataset Dataset codes. The codes for the dataset of the study.
(XLSX)

## Abbreviations

CPS: Child Protection Services
NPV: Negative Predictive Value
OPC: Out-of-hours Primary Care location
PPV: Positive Predictive Value
